# Healthy Food Intake Index (HFII) – Validity and reproducibility in a gestational-diabetes-risk population

**DOI:** 10.1186/s12889-016-3303-7

**Published:** 2016-07-30

**Authors:** Jelena Meinilä, Anita Valkama, Saila B. Koivusalo, Beata Stach-Lempinen, Jaana Lindström, Hannu Kautiainen, Johan G. Eriksson, Maijaliisa Erkkola

**Affiliations:** 1 Department of General Practice and Primary Health Care, University of Helsinki and Helsinki University Hospital, P.O. Box 20, Tukholmankatu 8 B, Biomedicum Helsinki, 00014 Helsinki, Finland; 2 Folkhälsan Research Centre, University of Helsinki, Helsinki, Finland; 3 Department of Obstetrics and Gynecology, University of Helsinki and Helsinki University Hospital, Helsinki, Finland; 4 Department of Obstetrics and Gynecology, South-Karelia Central Hospital, Lappeenranta, Finland; 5 Department of Chronic Disease Prevention, National Institute for Health and Welfare, Helsinki, Finland; 6 Department of General Practice and Primary Health Care, University of Eastern Finland, Kuopio, Finland; 7 Department of Food and Environmental Sciences, University of Helsinki, Helsinki, Finland

**Keywords:** Diet quality index, Dietary pattern, Gestational diabetes, Nutrition and pregnancy, Validation, Nordic nutrition recommendations

## Abstract

**Background:**

The aim was to develop and validate a food-based diet quality index for measuring adherence to the Nordic Nutrition Recommendations (NNR) in a pregnant population with high risk of gestational diabetes (GDM).

**Methods:**

This study is a part of the Finnish Gestational Diabetes Prevention Study (RADIEL), a lifestyle intervention conducted between 2008 and 2014. The 443 pregnant participants (61 % of those invited), were either obese or had a history of GDM. Food frequency questionnaires collected at 1st trimester served for composing the HFII; a sum of 11 food groups (available score range 0–17) with higher scores reflecting higher adherence to the NNR.

**Results:**

The average HFII of the participants was 10.2 (SD 2.8, range 2–17). Factor analysis for the HFII component matrix revealed three factors that explained most of the distribution (59 %) of the HFII. As an evidence of the component relevance 9 out of 11 of the HFII components independently contributed to the total score (item-rest correlation coefficients <0.31). Saturated fatty acids, monounsaturated fatty acids, polyunsaturated fatty acids, sucrose, and fiber intakes (among other nutrients) showed linearity across the HFII categories (*P* ≤ 0.030 for all nutrients tested); the higher the HFII, the closer the nutrient intake to the recommended intake level. Educational attainment (*P* = 0.0045), BMI (*P* = 0.0098), smoking (*P* = 0.007), and leisure time physical exercise (*P* = 0.038) showed linearity across the HFII categories. Intra-class correlation coefficient for the HFII was 0.85 (CI 0.79, 0.90).

**Conclusions:**

The HFII components reflect the food guidelines of the NNR, intakes of relevant nutrients, and characteristics known to vary with diet quality. It largely ignores energy intake, its components have independent contribution to the HFII, and it exhibits reproducibility. The main shortcomings are absence of red and processed meat component, and the validation in a selected study population. It is suitable for ranking participants according to the adherence to the NNR in pregnant women at high risk of GDM.

**Electronic supplementary material:**

The online version of this article (doi:10.1186/s12889-016-3303-7) contains supplementary material, which is available to authorized users.

## Background

Dietary analysis through single nutrients or foods is often too constricted [1]. The effect of a single nutrient may be insufficient due to interactions and inter-correlations with other nutrients, foods, or dietary patterns [2], and moreover, it may be too small to be identified. In contrast the cumulative effect of several foods, measured by dietary index for instance, addressing interactions and inter-correlations may become observable [3]. Few indices include only foods [4], and those that do [5, 6] tend to be rather concise and not specific enough for measuring subtle yet possibly essential elements, such as the quality of fat or the quality of carbohydrates, as well as the consumption of energy-rich/nutrient-poor foods. A nutrient-based index is rather burdensome for both the participant and the evaluator as it employs the use of a detailed diet record, a nutrient-calculation software, and a nutrient composition database. Food-based indices are necessary and useful when the calculations for precise nutrient intakes are infeasible, when the interest is in dietary choices rather than in nutrient intakes, or for a quick screening of a patient’s diet in health care settings.

Evidence based national and international nutrition recommendations provide a good basis for the construction of a diet quality index aimed to measure healthy eating [7]. Nordic Nutrition Recommendations (NNR) food-based guidelines are also applicable for pregnant women and adherence to these guidelines will be adequate to meet the nutritional needs of a healthy pregnant woman [8]. The NNR food guidelines [9] may also help in preventing gestational diabetes (GDM), since many of the food recommendations, namely high consumption of fruits, vegetables, whole-grains, fish and poultry, and low intakes of red and processed meat, and refined grains, have been associated with a lower risk of GDM [10–12]. Existing indices that are at least indirectly based on the NNR, such as The Baltic Sea Diet Score (BSDS) [13] and the Diet Quality Index, which is based on the Swedish Nutrition Recommendations 2005 (DQI-SNR) [14], are not solely food-based. The New Nordic Diet score (NND) [15], for example, takes into account issues beyond our aims, such as how environmentally friendly foods are and the locality of foods.

What led us to develop the Healthy Food Intake Index (HFII) was simply the lack of an appropriate food-based diet quality index for RADIEL and for other epidemiological studies where only food instead of nutrient intake data is available. This kind of index could be further adapted and validated for clinical use for diet quality screening. The aim of this study was to create a simple food-based diet quality index for measuring the adherence to the NNR food guidelines [9] and to validate the index among Finnish pregnant women at high risk of GDM.

## Methods

The study participants were part of the Finnish Gestational Diabetes Prevention Study (RADIEL), a multicenter lifestyle intervention study conducted in two Southern Finnish districts, namely the Helsinki Metropolitan area and Lappeenranta, between 2008 and 2014. The 727 participants were Finnish women with an elevated risk for GDM due to obesity (BMI ≥30 kg/m^2^), or a history of GDM, who were less than 20 weeks pregnant (*n* = 492) or were planning pregnancy (*n* = 235). Another criterion for inclusion in the study was that the participants were at least 18 years old. Exclusion criteria included diabetes diagnosed before pregnancy, medication influencing glucose metabolism, multiple pregnancy, physical disability, current substance abuse, severe psychiatric disorder and substantial communication difficulties. The data presented here is from the first trimester of pregnancy and it was collected during the woman’s first visit to the study nurse (between 2008 and 2014) during pregnancy. From the 235 women who were recruited before their pregnancy, 30 did not conceive within one year, 61 dropped out without getting pregnant or before the first visit to the study nurse during pregnancy, and 9 were excluded because they became pregnant with twins. From the 627 (86 %) participants who visited the study nurse in the first trimester of pregnancy, diet records were available for 485 (77 % of 627), but were inaccurately recorded for 4 women. The food frequency questionnaire (FFQ) was available for 624 participants, while for 586 participants it was complete enough to calculate their dietary score. The final number of participants that provided a HFII score and a food record was 443 (61 % of all) of whom 96 (22 %) were recruited before pregnancy and 347 (78 %) during pregnancy. For reproducibility analysis we used a subsample of control participants who did not have GDM diagnosis at the first trimester of pregnancy (*n* = 122). This study was conducted according to the guidelines laid down in the Declaration of Helsinki and all procedures involving human subjects were approved by The Ethics Committee of the Department of Obstetrics and Gynecology of Helsinki and Uusimaa Hospital District. Written informed consent was obtained from all participants.

### Data collection

#### Food intake data for constructing the Healthy Food Intake Index (HFII)

The data for the HFII were derived from the non-validated semi-quantitative food frequency questionnaires (FFQ). The validation and reproducibility process of the HFII also compensate the lack of validity assessment of the underlying FFQ; reproducibility of the HFII also reflects reproducibility of the FFQ, and comparison between the HFII and nutrient intakes from food records provides information on the validity of the FFQ. The participants filled in the FFQs at their first visit to the study nurse when they were between 6 and 18 weeks pregnant, with a mean gestational age of 12.5 weeks (SD 1.9). For the reproducibility analysis we also used the FFQs from weeks 22 to 30 (mean gestational age 26 (SD 1.9)), for calculating the HFII_2trimester_. The FFQs included 48 foods with 7 frequency options ranging from “less than once a week or never” to “more than 4 times per day”. Twenty-one foods from the FFQ were used for the HFII. In addition, there were 9 qualitative questions in the FFQ, of which two on the quality of fats, namely fat spreads and cooking fat, and these were used to develop the HFII. The options for fat spreads were margarine, low-fat margarine, sterol margarine, butter-oil mix, or butter, and those for cooking fats were vegetable oil, margarine, liquid margarine, baking margarine, butter-oil mix, or butter.

#### Components of the Healthy Food Intake Index (HFII)

The components for the HFII were selected to reflect the content of food-based guidelines of the Nordic Nutrition Recommendations (NNR) [9]. The main food-based guidelines of the HFII are presented in the first column of Table 1. The HFII comprised 11 components and covered the following food groups; vegetables, fruits and berries, high-fiber grains, fish, low-fat milk, low-fat cheese, cooking fat, fat spread, snacks, sugar-sweetened beverages, and fast food. The HFII is a sum of the 11 components, and the scores range between 0 and 17.

**Table 1:** Components and scoring of the Healthy Food Intake Index (HFII) validated among Finnish pregnant women at high risk of gestational diabetes

Main guidelines in NNR	HFII component	Included foods	Intake freq.	Score	Principle for cut-off
1. Limit:					
Beverages and foods with	Snacks	candy, chocolate, pastries,	≤4 x / wk	2	tertiles
added sugar or salt		chips, ice cream	5–6 x / wk	1	
			≥1 x / d	0	
	Sugar-sweetened	sugar-sweetened soft drink	<1 x / wk	1	median
	beverages	and sugar-sweetened juice	≥1 x / wk	0	
	Fast food	hamburgers ja pizza	<1 x / wk	1	median
			≥1 x / wk	0	
Processed and red meat	-				
Alcohol	-				
2. Exchange:					
Refined cereals to	High-fiber grains	dark bread, brown rice and	≥3 x / d	2	FNR + CP
→ whole grain cereals		pasta, porridge	1–2 x / d	1	
			<1 x / d		
Butter to	Cooking fat	vegetable oil/margarine		1	NNR
→ vegetable oils		/liquid margarine/no fat			
		butter, butter-oil mix		0	
		/baking margarine			
Butter based	Fat spread	margarine, low-fat margarine		2	NNR
bread fat spreads to		sterol margarine, or if		1	
→ oil-based fat		options from more than			
spreads		one category chosen			
		butter or butter-oil-mix/no spread		0	
		/no spread			
High-fat dairy to	Low-fat cheese	fat percentage ≤17 %		1	NNR
→ low-fat dairy		fat percentage >17 % / no cheese		0	
	Low-fat milk	only low-fat milk (≤1 % fat)		2	NNR
		both low-fat and full fat milk		1	
		full fat milk or no milk at all		0	
3. Increase:					
Vegetables, fruits,	Vegetables	vegetables, legumes	>2 x / d	2	FNR + CP
and berries			1–2 x / d	1	
			<1 x / d	0	
	Fruits and berries		≥1 x/d	1	FNR + CP
			<1 x / d	0	
Fish and seafood	Fish		≥1 x / wk	2	FNR + CP
			<1 x / wk	0	
Nuts and seeds	-				
Total HFII, maximum score				17	

*NNR* Nordic Nutrition Recommendations 2012 [9], *FNR* Finnish Nutrition Recommendations 2014 [16], *CP* Consensus Panel decision

The HFII components snacks, sugar-sweetened beverages, fast food, vegetables, and fruits and berries, included all the foods that the FFQ provided for those food groups. The foods not incorporated into the index, such as potato, soy products, internal organs, coffee or tea, were not relevant considering the content of the NNR. Within the main 12 NNR food groups, three groups were missing from the HFII, namely meat, alcohol, and nuts and seeds (Table 1). The different types of meats (red / white and processed), and nuts (salted / non-salted) were impossible to separate from each other, because of the way the questions had been laid out in the FFQ. Alcohol intake in the current population was marginal, and was therefore not included in the HFII. Liquid dairy products included only milk, because reliable categorization of yoghurts, based on their fat content, was not possible since this information had not been collected. The high-fiber grain -category did not include breakfast cereals as we could not classify them according to their sugar and fat content.

#### Scoring of the Healthy Food Intake Index (HFII)

The scoring of the HFII is presented in Table 1. The score was set to reflect the food-based guidelines of the NNR where feasible. Where NNR did not provide unequivocal or numerical recommendations, we applied Finnish Nutrition Recommendations (FNR; based on the NNR) [16] and a consensus agreement in a panel of nutrition experts was applied in the cut-offs.

Scores for consumption of cheese and milk were assigned based on their fat content. Participant’s choice between high SFA vs. low SFA content was applied for cooking fat and fat spread components. For high-fiber grains, vegetables, fruits and berries, and fish the cut-offs were set according to the FNR recommendation on the number of portions per day. For the rest of the components (fast food, snacks, and sugar-sweetened beverages) numeral recommendations were absent from the NNR and the FNR, leading to the cut-offs being set according to medians and tertiles of the frequency of use in the study population.

#### Weighting of the HFII components

Each score component was assigned a maximum score value of either 1 or 2, based on a priori assumption of the relative importance of the category for the overall diet quality. Based on findings in the latest national Findiet survey [17], fat used as spread was considered more important source of total and saturated/unsaturated fats compared to cooking fat. Similarly, type of milk was considered to contribute more to total dairy fat intake than cheese. Snacks component includes more food groups than sugar-sweetened beverages and fast food and was, therefore, assigned maximum of 2 points whereas maximum of one point was assigned for both sugar-sweetened beverages and fast foods.

#### Nutrient intake data

The three-day estimated food records were used for assessing criterion validity, i.e. as a reference method for the validity of the HFII. The food records were collected prior to the FFQ. The participants were asked to record all foods and beverages they consumed during three consecutive days (2 weekdays and 1 weekend day) using household measures or weights, where applicable, and submit the food record at their first visit to the study nurse. Two trained nutritionists assessed and entered the data into the nutrient-calculation software AivoDiet, version 2.0.1.5 (Aivo Finland Oy). The Finnish National Institute for Health and Welfare (www.fineli.fi) provided the food composition database used by the software. A more detailed description of the collection and handling of the food records have been presented in our previous paper [18].

#### Demographic characteristics and anthropometric measures

The participants filled in a questionnaire on their health, lifestyle habits, and history of pregnancies and pregnancy-related issues. The questionnaire also gathered information on age, basic education, highest level of education, smoking (yes/no), and time used for physical exercise. The number of years of education was calculated based on the reported basic and highest education. Leisure time physical activity was queried as time per week used for physical exercise during the last month. Body mass index (BMI) was calculated from the weight and height measured at the woman’s first visit to the study nurse during pregnancy. The validation, component-analysis and reproducibility-analysis protocol are presented in Table 2.

**Table 2:** Validation protocol of the Healthy Food Intake Index (HFII) among pregnant Finnish women at high risk of GDM: type of validity and HFII components, and adopted approach for evaluation

Reproducibility 1. Does the HFII_1_ measured at 1st trimester adequately agree with the HFII_2_ measured at 2nd trimester?	1. Kappa coefficients between 1st and 2nd pregnancy trimesters’ HFII components, intra-class correlation coefficient for 1st and 2nd trimesters’ total HFII.
Content validity 2. Do the index components cover all the food groups of the underlying recommendations of healthy diet (NNR)?	2. Comparing the content of the HFII with NNR.
Construct validity 3. Does the HFII create variation in the population? 4. Is scoring independent from energy intake? 5. Does the HFII have multidimensional construct and what are the dimensions it measures?	3. Item analysis of the HFII components: corrected item correlation and item mean. 4. Energy intake from food records. Comparisons between the HFII categories: general linear models 5. Iterated principal factor analysis for the HFII components matrix.
Components 6. Do the components have independent roles within the HFII? 7. Which components provide the highest and lowest scores?	6. Corrected item correlation 7. Item mean
Criterion validity 8. Does a linear trend in nutrient intake exist across the HFII categories, or index component categories? 9. Does the HFII distinguish between groups with known differences in diet quality?^a^ a. Age b. Education c. BMI d. Smoking e. Physical activity	8. Statistical comparisons between the HFII categories: bootstrap-type general linear models with the appropriate contrast 9. Same as 8^.^

*GDM* gestational diabetes, *NNR* Nordic Nutrition Recommendations 2012 [9]; ^a^[25, 26, 29, 27, 47]

### Statistical methods

Descriptive results are expressed as percentages, mean or median, standard deviation (SD), or interquartile range (IQR). Differences between the participants with available dietary data (who were included in the further analysis) and participants without available dietary data (excluded from the further analysis) were tested by Chi-square test, Student’s *t*-test, Mann–Whitney *U*-test or Fisher’s exact test, depending on the distribution of the variables. Construct validity was studied by iterated principal factor analysis with varimax rotation for the HFII component matrix of polychoric correlations. Item analysis of the HFII components was performed by analyzing item discriminating power (corrected item correlation) and item difficulty (item mean), depicted by explanatory data analysis. Corrected item correlation was estimated using polychoric or polyserial correlations. In order to test how the HFII was associated with nutrient intakes and with characteristics associated with a healthy diet, the HFII was divided into three categories by setting cut-off limits at ± one deviation from the mean (from here onwards referred to as HFII categories). Statistical comparisons between the categories were performed using bootstrap-type general linear models with the appropriate contrast. The bootstrap method was used when the theoretical distribution of the test statistics was unknown or when it violated the assumptions (e.g. non-normality). The normality of the variables was tested using the Shapiro-Wilk W test. Reproducibility of the HFII components was evaluated from the HFII_1trimester_ and the HFII_2trimester_ using the weighted Kappa coefficient, and that of the total HFII using intra-class correlation coefficient (ICC) with one-way random-effects model. Thresholds for the Kappa coefficients were considered according to Landis & Koch [19]: 0 = less than chance agreement; 0.01–0.20 = slight agreement; 0.21–0.40 = fair agreement; 0.41–0.60 = moderate agreement; 0.61–0.80 = substantial agreement; 0.81–0.99 = almost perfect agreement. Stata 13.1, StataCorp LP (College Station, TX, USA) statistical package was used for the analyses.

## Results

The participants with available dietary data had less frequently a history of GDM (49 % vs. 59 %, *p* = 0.03), and higher educational attainment (14.4 vs. 14.1 years, *p* = 0.04) compared to participants without available dietary data. No other differences were observed (results not shown). The average age of the 443 participating pregnant women with a high risk of GDM was 32.4 years (SD 4.5). Of this number, 140 (31 %) were nulliparous, 85 (19 %) had no history of GDM, and 218 (49 %) had a history of GDM. The average number of years of education was 14.4 (SD 4.7), and the mean BMI at the first study visit was 31.9 kg/m^2^ (SD 5.7). The number of smokers among the participants was 22 (5 %) and the median time used for physical exercise was 60 min/week (IQR 30; 140).

The mean score (HFII) among pregnant women at high risk of GDM was 10.2 points (SD 2.8), with a range of 2 to 17. Three participants (0.7 %) had the highest possible score of 17. The distribution of the HFII did not differ from normality (*P* = 0.41) (Additional file 1) but was slightly negatively skewed (*P* = 0.081).

### Content validity

The Healthy Food Intake Index (HFII) covered all the food groups addressed in the NNR food guidelines, apart from alcohol, processed and red meat, and nuts (Table 1).

### Criterion validity

**Fig. 1:**
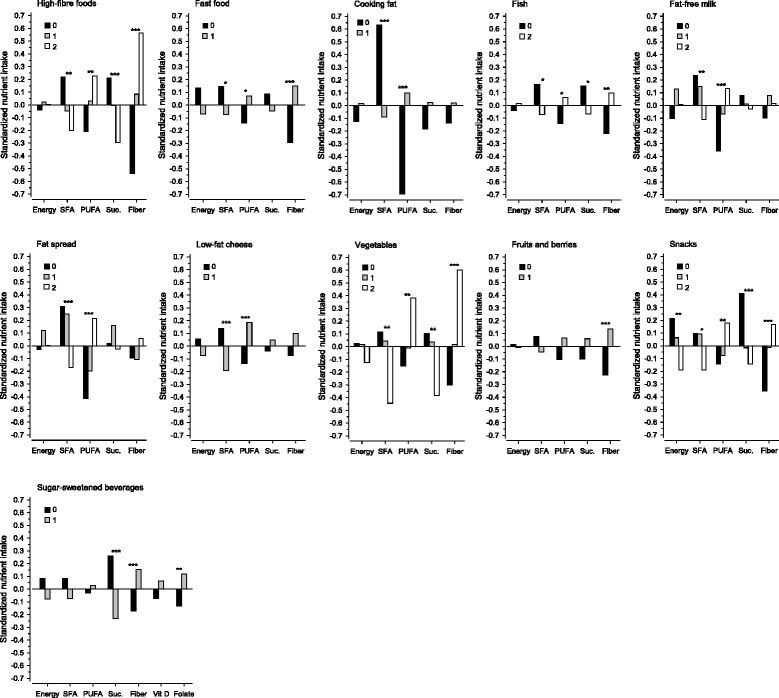
Intake of nutrients in categories of the Healthy Food Intake Index (HFII) components among pregnant Finnish women at high risk of gestational diabetes. Differences tested by bootstrap-type general linear models with linear contrast. Statistically significant at level *P < 0.05, **P < 0.01, ***P < 0.001. Suc., Sucrose

The energy-adjusted intakes of the energy-yielding nutrients, including saturated fatty acids (SFA), monounsaturated fatty acids (MUFA), and polyunsaturated fatty acids (PUFA), and the intakes of fiber, sucrose, vitamins A, D, E, and folate, showed linearity across the three total HFII categories (Table 3). The intake of energy was not statistically significantly associated with the three total HFII categories (*P* = 0.24), which is an indication of the HFII measuring diet quality instead of diet quantity. The linearity of the nutrient intakes in the HFII-component categories is presented in Fig. 1. The components that provided the highest scores (fish, low-fat milk, fat spread, and snacks) as well as the component with the lowest score (low-fat cheese), all reflected the intake levels of SFA and PUFA.

**Table 3:** Intake of nutrients among pregnant Finnish women at high risk of gestational diabetes by Healthy Food Intake Index (HFII) categories^a^

	HFII						
	0–7		8–12		13–17		
	Mean	SD	Mean	SD	Mean	SD	*P*-value^b^
Energy	1943	551	1918	405	1856	411	0.24
Carbohydrate E %	42.8	7.4	45.3	5.8	46.1	5.7	0.002
Protein E %	17.5	3.7	17.8	3.0	19.0	2.8	0.001
Fat E %	36.9	7.5	33.5	5.8	30.9	5.6	< 0.001
SFA % from total fat	38.9	5.3	36.6	5.4	33.6	5.4	< 0.001
MUFA % from total fat	33.5	3.0	34.4	2.7	34.9	3.0	0.0017
PUFA % from total fat	15.5	3.3	17.4	3.8	19.6	4.1	< 0.001
Sucrose E %	9.6	4.6	8.9	3.6	7.4	3.3	< 0.001
Dietary fiber g/MJ	2.5	0.9	2.9	0.9	3.5	0.8	< 0.001
Vitamins:							
C mg/MJ	14.9	10.7	17.7	7.9	20.4	8.5	< 0.001
E mg/MJ	1.3	0.3	1.3	0.3	1.4	0.3	< 0.001
A μg/MJ	89.6	45.0	92.9	44.1	103.3	40.2	0.03
D μg/MJ	0.8	0.4	0.9	0.5	1.1	0.8	< 0.001
Folate μg/MJ	32.4	9.4	35.6	8.4	40.0	6.9	< 0.001

^a^Cut-off limits ± one deviation from the mean. ^b^Tested by bootstrap-type general linear models with linear contrast

Educational attainment (years of education) (*p* = 0.0045) and physical activity (*p* = 0.038) showed positive linearity across the three HFII categories. BMI (*p* = 0.0098) and smoking (0.0079) showed negative linearity across the three HFII categories whereas age (*p* = 0.18) did not show a statistically significant trend.

### Construct validity

Factor analysis for the HFII component matrix revealed that within all the HFII components (Table 4), there were three distinct factors that explained most of the variation (59 %) within the score. According to the food group loadings, three major factors were identified. The first was characterized by high loadings for components cooking fat, fat spread, low-fat cheese, and low-fat milk (named as Fat factor). The second was characterized by high loadings for high-fiber grains, vegetables, fruits and berries, and fish (named as Healthy foods). The third factor was characterized by high loadings for snacks, sugar-sweetened beverages, and fast food (named Unhealthy foods). This provided information on correlational structure of the components and that the HFII measured meaningful dimensions of diet.

**Table 4:** Factors^a^ among the components of the Healthy Food Intake Index (HFII) among pregnant Finnish women at high risk of gestational diabetes

Component	“Fats”	“Healthy foods”	“Unhealthy foods”
Low-fat cheese	0.67		
Low-fat milk	0.66		
Fat spread	0.70		
Cooking fat	0.79		
Fish		0.66	
Vegetables		0.81	
Fruit and berries		0.60	
High-fiber grains		0.45	
Snacks			0.72
Fast food			0.80
Sugar-sweetened beverages			0.58

^a^Explanatory factor analysis with varimax-rotated factor loadings. Factor loadings with values < 0.45 not shown

### Components of the HFII

**Fig. 2:**
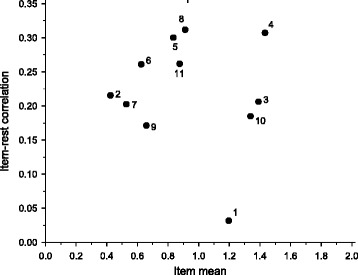
Item-analysis of the components of the Healthy Food Intake Index (HFII) among pregnant Finnish women at high risk of gestational diabetes. X-axis represents the mean score that the participants got from the component, and the Y-axis represents the correlation of a component to the rest of the components. The dashed line represents the mean score of all the components. 1 = Snacks, 2 = Low-fat cheese, 3 = Fish, 4 = Low-fat milk, 5 = Vegetables, 6 = Fruits and berries, 7 = Sugar-sweetened beverages, 8 = High-fiber grains, 9 = Fast food, 10 = Fat spread, 11 = Cooking fat

The components of the HFII that provided the highest scores were milk, fish, fat spread, and snacks (Fig. 2). The component that provided the lowest scores was low-fat cheese. All components had item-rest correlation coefficients less than 0.31 with the rest of the components, snacks having the lowest item-rest correlation coefficient (0.03). High-fiber grains and vegetables had item-rest correlation coefficients of similar quantity and item means close to each other, which prevents us from interpreting whether they act independently in the HFII. The component scores of high-fiber grains and vegetables, however, reflected nutrient intakes of different magnitude which suggests that they had at least some independency. All components independently contributed to the total score and created variation within the study population, but for high-fiber grains and vegetables independency may have been weak.

### Reproducibility

Weighted Kappa coefficients between the HFII_1trimester_ and the HFII_2trimester_ components ranging from 0.41 to 0.69 (Table 5) suggest moderate to substantial agreement [19]. The ICC between the HFII_1trimester_ and the HFII_2trimester_ was 0.85 (95 % CI 0.79 to 0.90). The repeatability coefficient of the HFII was 3.7 (95 % CI 3.3–4.3) units, meaning there was a 95 % chance that two measurements will differ by less than 3.7.

**Table 5:** Agreement of the HFII_1trimester_- and the HFII_2trimester_-components and Kappa coefficients between them

	Observed agreement % (95 % CI^a^)	k (95 % CI)
Snacks	68 (58, 75)	0.56 (0.42, 0.70)
Low-fat cheese	79 (70, 84)	0.56 (0.42, 0.71)
Fast food	78 (69, 84)	0.44 (0.26, 0.61)
Low-fat milk	81 (75, 89)	0.65 (0.48, 0.79)
High fiber grains	67 (60, 75)	0.60 (0.45, 0.72)
Fish	75 (66, 81)	0.43 (0.26, 0.60)
Fruits and berries	76 (70, 84)	0.48 (0.32, 0.64)
Cooking fat	92 (87, 98)	0.67 (0.49, 0.86)
Fat spread	80 (74, 88)	0.69 (0.55, 0.81)
Vegetables	72 (65, 80)	0.59 (0.45, 0.72)
Sugar-sweetened beverages	70 (63, 80)	0.41 (0.25, 0.57)

^a^Confidence intervals (95 % CI) were obtained by bias-corrected and accelerated bootstrapping (5000 replications)

## Discussion

The study evaluated validity and reliability of the Healthy Food Intake Index (HFII) in a population of pregnant Finnish women who had a high risk of GDM and were participating in the Finnish Gestational Diabetes Prevention Study RADIEL.

The HFII succeeded in reflecting the level of adherence to the recommended nutrient intake levels of the NNR. The study showed that when moving from a lower to a higher HFII category, the nutrient density, apart from fat was higher. This result was similar to the results of a study of the Baltic Sea Diet Score (BSDS) [13], Diet Quality Index – Swedish Nutrient Recommendations 2005 (DQI-SNR) [14] and the New Nordic Diet score (NND) [15]. An advantage compared to the BSDS [13] was that the HFII additionally reflected the intake of sucrose. High intake of sucrose may contribute to the development of gestational diabetes [20] and overweight [21]. The highest category in the current study did not reflect sufficient intake of folate and vitamin D as per the recommended nutrition density of the NNR. This should be taken into account when using the HFII. Sufficient intake of folate is especially important in early pregnancy [22] and the recommended nutrient intake level of the NNR [16] is higher for pregnant women than for the general population. The intakes of folate and vitamin D in the Finnish population have been below the NNR’s recommended daily intakes [23, 24] and supplementation is recommended for all pregnant women. The finding that the HFII score was rather independent of energy intake, indicates that the HFII does, to a large extent, ignore the quantity of food intake. The unwanted consequence of providing higher scores for eating more food in general, was in part reduced by the components that measured intake frequencies and habitual choices rather than amounts of intake. Being able to avoid energy-adjustment contributes considerably to the HFIIs usability because it rules out the need for detailed dietary data. Further evidence for the capacity of the HFII to rank participants according to the healthiness of the diet was provided by the parallel association of the HFII with the characteristics known to associate with the healthiness of diet [25–29]. The reproducibility measures of the current study were similar to the few earlier reproducibility studies on food intake during pregnancy [30, 31]. In the light of studies of dietary changes through pre-pregnancy to pregnancy, assessing reproducibility during pregnancy seems reasonable. Cuco and her colleagues [32] as well as Crozier and her colleagues [33] both found little overall change in dietary patterns from pre-pregnancy trough early to late pregnancy.

The factors identified as “fat”, “healthy”, and “unhealthy”, show that the HFII captured reasonable aspects of diet; such food choices have also occurred jointly in other studies [2, 34, 35]. The HFII-components nevertheless had independent roles within the total HFII, which was supported by the low item-rest correlation coefficients. A very low correlation with the rest of the index, however, may suggest a flaw within the component. One such component which we decided to retain in the current study because it linearly reflected the intakes of six important nutrients was snacks. Its low scores reflected the intakes further from and the high scores reflected intakes closer to the recommended nutrient intake levels of the NNR. Lower item-rest correlation of snacks may, however, indicate cut-off limits that are not comparable to the other components in reflecting a healthy diet. Re-setting the cut-off limits for snacks could improve the internal consistency of the HFII and should be considered.

The HFII distinguished whole-grain products from refined grains, and fatty milk from skimmed milk, as proposed by Waijers and co-workers [4]. Excluding red and processed meat may have impaired the accuracy of the HFII in reflecting all aspects of a healthy diet, because red and processed meat may increase the risk for cancer, cardiovascular disease, type 2 diabetes [20], and gestational diabetes [11]. In future studies, a component of red and processed meat could be added. Subjective interpretations in scoring, setting cut-off limits, and weighting, all common problems of indices [2], may have resulted in some suboptimal scorings, for example in the case of the snacks component. A more detailed FFQ could have provided more accurate cut-offs for whole grain foods, vegetables, fruits and berries, and fish. Population-based cut-offs may perform differently in other populations, and, therefore, scores between different populations may not be comparable with each other.

As a food-based index, the HFII takes into account the complexity of foods, and makes detailed nutrient-intake data and the use of major resources unnecessary [36]. Its components, nutrient intakes and characteristics in its categories compare well with the findings from the BSDS [13], the DQI-SNR [14], and the NND [15]. A major advantage of the HFII compared to many other indices is its independency of energy intake [13–15, 37]. Other notable advantages of the HFII are its simplicity and the approved multi-perspective evaluation including analysis of the relevance of its components and their interrelationships, something that the majority of index validation studies fail to recognize [4].

One weakness of the current study was that the FFQ underlying the HFII was not validated, which may have affected the accuracy of the scores. This, however, is at least partly compensated by testing validity and reliability of the HFII. Comparison of food record, that measures short term intake [38], with HFII, that measures habitual intake, may have attenuated the association between the HFII and the nutrient intakes [39]. A replicate food record could have improved the evaluation [40]. Obesity, and pregnancy are factors that result in under-reporting of dietary intake [41, 42] and therefor constitute a challenging group for dietary assessment. Thus, the scores may be over-estimates. However, because the HFII proved to be independent of energy intake, it may be less sensitive to under-reporting. Available dietary data seemed to add to the selection bias, which was already prevalent because of the study design. Obese pregnant women and women with GDM history do not represent general pregnant population, but cover a wide proportion of it; approximately one third of Finnish [43] women at childbearing age are either overweight or obese, the prevalence of GDM being approximately 13 % [44, 45]. For more generalizable results validation in a more general population, however, is required. Since nutrition recommendations in Western countries tend to have similar main principles [46], the HFII could be used in other Western countries with minor adaptions in the food groups and with re-evaluation. Similar food guidelines apply for pregnant and non-pregnant populations [9] so the HFII could be also suitable for other adult populations. Based on the current study the HFII can be used among pregnant women in countries with similar food consumption and food guidelines to Finland, namely the Nordic countries. For further evaluation studies in different populations we have now provided a detailed scoring system and thorough validation protocol to be applied.

## Conclusions

Despite the shortcomings of the HFII, it covered all relevant food groups mentioned in the food guidelines of the NNR, excluding red and processed meat. Secondly, the nutrient intakes came closer to the recommended intake of the NNR for all macronutrients and all vitamins and minerals measured when stepping towards the higher HFII categories. Thirdly, all components had a contribution to the HFII. Fourthly, demographic characteristics varied across the HFII categories meaningfully. Thus, the HFII can be used without detailed dietary data or energy-adjustment in studies for ranking the participants according to the level of adherence to the food-guidelines of the Nordic Nutrition Recommendations among overweight and obese pregnant women or pregnant women with a history of GDM. It has also great potential to be adapted in other adult populations in countries with similarities to Finland in dietary patterns.

## Abbreviations

BMI, body mass index; BSDS, The Baltic Sea Diet Score; DQI-SNR, Diet Quality Index – Swedish Nutrient Recommendations 2005; GDM, gestational diabetes; HFII, Healthy Food Intake Index; NND, new nordic diet score; NNR, nordic nutrition recommendations

## Additional file

Additional file 1: Distribution of the scores of the Healthy Food Intake Index (HFII) among pregnant Finnish women at high risk for gestational diabetes. (DOCX 22 kb)
