# Supplementary material for: Healthy Food Intake Index (HFII) – Validity and reproducibility in a gestational-diabetes-risk population
Source: BMC Public Health. 2016 Jul 30;16:680. doi: 10.1186/s12889-016-3303-7 (PMC4967513; doi:10.1186/s12889-016-3303-7)
Supplement: Additional file 1: — Distribution of the scores of the Healthy Food Intake Index (HFII) among pregnant Finnish women at high risk for gestational diabetes. (DOCX 22 kb) [file 12889_2016_3303_MOESM1_ESM.docx]

HFII-score

Additional file 1. Distribution of the scores of the Healthy Food Intake Index (HFII) among pregnant Finnish women at high risk for gestational diabetes.
